# Common Methods for Handling Missing Data in Marginal Structural Models: What Works and Why

**DOI:** 10.1093/aje/kwaa225

**Published:** 2020-10-15

**Authors:** Clémence Leyrat, James R Carpenter, Sébastien Bailly, Elizabeth J Williamson

**Keywords:** complete cases, inverse probability weighting, last observation carried forward, missingness pattern approach, multiple imputation, propensity score, time-varying confounding

## Abstract

Marginal structural models (MSMs) are commonly used to estimate causal intervention effects in longitudinal nonrandomized studies. A common challenge when using MSMs to analyze observational studies is incomplete confounder data, where a poorly informed analysis method will lead to biased estimates of intervention effects. Despite a number of approaches described in the literature for handling missing data in MSMs, there is little guidance on what works in practice and why. We reviewed existing missing-data methods for MSMs and discussed the plausibility of their underlying assumptions. We also performed realistic simulations to quantify the bias of 5 methods used in practice: complete-case analysis, last observation carried forward, the missingness pattern approach, multiple imputation, and inverse-probability-of-missingness weighting. We considered 3 mechanisms for nonmonotone missing data encountered in research based on electronic health record data. Further illustration of the strengths and limitations of these analysis methods is provided through an application using a cohort of persons with sleep apnea: the research database of the French Observatoire Sommeil de la Fédération de Pneumologie. We recommend careful consideration of 1) the reasons for missingness, 2) whether missingness modifies the existing relationships among observed data, and 3) the scientific context and data source, to inform the choice of the appropriate method(s) for handling partially observed confounders in MSMs.

## Abbreviations

CC: complete-case
CPAP: continuous positive airway pressure
IPMW: inverse-probability-of-missingness weighting
LOCF: last observation carried forward
MAR: missing at random
MCAR: missing completely at random
MI: multiple imputation
MPA: missingness pattern approach
MSM: marginal structural model
OSFP: Observatoire Sommeil de la Fédération de Pneumologie

Although randomized trials are the gold standard for establishing causal effects of treatments and nonpharmacological interventions on health outcomes, observational data are increasingly being used for causal inference (1). The enormous potential offered by the wealth of routinely collected medical data available and the need for real-world evidence to assess the efficacy and safety of treatments have contributed to this phenomenon.

These routinely collected data typically have a longitudinal structure, following people over time, allowing the measurement of dynamic treatment patterns, including treatment switching or delay to treatment initiation. Patients with chronic conditions often have a nonlinear treatment history: Treatment prescription might be updated based on the occurrence of new health events, changes in individual factors, or side effects induced by previous treatments. The newly prescribed treatment might, in turn, affect future health events and individual factors, themselves potentially associated with the outcome of interest (2). In such settings, specific statistical methods are required to account for confounding bias induced by time-varying variables (3). Indeed, adjusting for the confounders and treatment history is not sufficient, and often leads to biased estimates of the causal treatment effects (4). This is because the effect of a treatment received at a specific time on the outcome is mediated by subsequent treatments. Propensity scores—the individual probabilities of receiving the treatment of interest conditionally on individual characteristics—have been extended to situations with time-varying treatment and confounders (5), with scores estimated at each time point (6). The cumulative product of the inverse of these scores over time can be used as a weight to account for confounding in the estimation of the treatment effect in a marginal structural model (MSM) (7). This method of adjusting for time-varying confounders is the most common in practice, by far (8).

A challenge when analyzing observational data is incomplete confounder information. In routinely collected data, missingness is particularly prevalent among covariates. This can happen if some information is not recorded at a given time point or the frequency of the measurement varies from one patient to another (e.g., asynchronous medical visits (9)). This might jeopardize the validity of the results if the issue is ignored in the analysis, depending on the underlying missingness mechanisms. In practice, despite the STROBE recommendations to report the amount of missing data and the way in which they are handled (10) in observational studies, reporting is often suboptimal. A review of reporting of missing exposure data in longitudinal cohort studies showed that 43% of identified publications adhered to these guidelines (11). Importantly, when the method for handling missing data was reported, it was often done using inadequate methods. Although several methods for handling missing data on covariates have been used in the context of time-varying exposures (11), the most common approaches—complete-case (CC) analysis and last observation carried forward (LOCF)—have been criticized. Use of more complex approaches such as multiple imputation (MI) or inverse-probability-of-missingness weighting (IPMW) have been suggested, but their performance is yet to be fully explored. Another promising approach, which has not been extended to the context of MSMs, is the missingness pattern approach (MPA).

To our knowledge, there are no published guidelines on the choice of methods for handling missing confounder data in MSMs. Published studies on missing data in MSMs focused on missing data in the exposure (11, 12) or compared the performance of a few methods only (13, 14). Moodie et al. (14) compared the use of IPMW and MI, finding that MI outperformed IPMW, but they did not investigate the performance of MPA and LOCF. Moreover, only 1 covariate and 2 time points were considered, limiting the generalizability of the results. Vourli and Touloumi (15) investigated the performance of MI, IPMW, and LOCF but reached opposite conclusions in their setting, finding that IPMW usually performed better than MI. This might be explained by the omission of the outcome from the imputation model. A recent plasmode simulation (13) suggested superiority of MI over IPMW, but surprisingly, CC analysis was the least biased. A limitation of these published studies is their focus on missingness mechanisms described under Rubin’s taxonomy of missing data (16); this taxonomy may be too restrictive to describe complex missingness scenarios encountered in routinely collected data (17).

Our aims in this paper are, first, to provide an overview of existing methods for handle missing data on confounders in MSMs and, second, to recommend practical guidelines. These guidelines will rely on the understanding of the assumptions and missingness mechanisms under which these methods are valid. We focus on situations where some variables are not recorded during the visit, rather than missing data introduced because of sparse follow-up. These 2 scenarios differ in terms of both underlying missingness mechanisms and required statistical methods. The challenges of sparse follow-up have been discussed by Mojaverian et al. (9) and Kreif et al. (18). We present the design and results of a simulation study comparing the performance of CC analysis, LOCF, MI, IPMW, and MPA in handling partially observed confounders under common missingness mechanisms encountered in observational studies. Finally, we illustrate the implementation of these methods by investigating the impact of treatment compliance on sleepiness in patients with sleep apnea.

## MSMs AND THE ISSUE OF MISSING DATA

### Causal inference in the presence of time-varying treatment and confounders

When time-varying confounding occurs, standard regression approaches fail because of treatment-confounder feedback (19), even when models are adjusted for past treatment and confounders (3). MSMs were developed (7) to estimate causal effects in this setting. MSMs rely on an extension of inverse-probability-of-treatment weighting, a propensity score approach, for multiple time points. Details about this framework and underlying assumptions for a single time point are provided in Web Appendix 1 (available at https://doi.org/10.1093/aje/kwaa225). Similar to propensity score approaches, MSMs are a 2-stage process. In the first stage (19), weights—based on the inverse of the probability of a patient’s receiving the treatment they actually received—are estimated to create a pseudopopulation in which treatment and confounders are independent. In the second stage, a weighted regression (using the weights derived in the first stage) including only the treatment history can be used to obtain an estimate of the causal effect of the treatment regimens of interest. Under the assumptions of no interference, consistency, exchangeability, and positivity extended to time-varying settings, and assuming that the model used to obtain the weights is correctly specified, MSMs lead to unbiased estimates of the marginal causal effect of the treatment regimen.

In practice, the weights can be estimated using pooled logistic regression (6), in which each person-time interval is considered as an observation. This pooled logistic regression model must include the confounders and their relevant interactions to ensure that the distributions of confounders are balanced between treatment groups in the weighted pseudopopulation at each time point. Further details on the implementation of MSMs can be found in Web Appendix 2.

### Missing data in MSMs: mechanisms and methods

The choice of an appropriate missing-data method relies on the characterization of the missingness patterns and the missingness mechanisms. The missingness patterns simply define which values of the covariates are observed and which are missing. For example, if the data set contains only 2 time-fixed covariates, *L*_1_ and *L*_2_, there are 4 missing data patterns: *L*_1_ and *L*_2_ can both be observed, *L*_1_ and *L*_2_ can both be missing, *L*_1_ can be observed and *L*_2_ missing, or *L*_1_ can be missing and *L*_2_ observed. Similarly, if *L*_1_ and *L*_2_ are measured at 2 time points, there are 16 patterns. Some methods for handling missing data, such as multiple imputation, apply to any pattern of missing data; other methods apply only to specific structures of missing data, the most common being the monotone missing-data pattern. In longitudinal data, monotone missingness patterns occur when, once a patient has a missing observation at 1 time point, values for all subsequent time points are also missing. This is typically what happens when patients are lost to follow-up. Whereas methods based on inverse weighting have been proposed to address this type of missing data in MSMs, there is no guidance on how to handle arbitrary patterns (not monotone) in MSMs. This is, however, the most common pattern found in routinely collected data where data are not collected for research purposes, and the quality of recording may vary from one visit to another.

Little and Rubin’s classification (16) is often used to classify the missing data as being 1) missing completely at random (MCAR), when the probability of data being missing does not depend on the observed or unobserved data; 2) missing at random (MAR), if the probability of data being missing does not depend on the unobserved data, conditional on the observed data; or 3) missing not at random (MNAR), if the probability of data being missing depends on the unobserved data, even after conditioning on the observed data (20).

A variety of methods for handling missing data on covariates have been used in the context of time-varying exposures (11). The most common approach is CC analysis, in which only patients with a complete record for all of the covariates are included in the analysis. Another simple and popular approach is LOCF: When a measurement is missing for a given patient, the most recent past value observed for that patient is used to impute the missing value. MI uses relationships existing among the observed variables to draw plausible values multiple times for the missing data; the standard error of the treatment effect estimates accounts for the uncertainty in these predictions. In MSMs, Robins et al. (7) proposed using censoring weights to account for patients lost to follow-up. Complete cases are reweighted by the inverse of their probability of remaining in the study. Loss to follow-up can be viewed as a missing-data problem, and therefore these weights can be accommodated to account for missing data. This method is called IPMW. Another promising approach for handling partially observed confounders that should be extended to MSMs is the MPA. The MPA has been proposed for the estimation of propensity score weights in studies with a single time point (17). The sample is split into subgroups of patients having missing information on the same set of covariates, and the weights are derived in each subgroup from the covariates available in that pattern. More details on these methods are presented in Web Appendix 3. The approaches rely on different assumptions; their validity depends on the missingness mechanisms in the data at hand. These assumptions, along with the strengths and limitations of each method, are summarized in Table 1.

**Table 1:** Characteristics of 5 Missing-Data Methods for Partially Observed Time-Varying Confounders

**Method**	**Missing Data on…**	**Assumptions**	**Unbiased in MSMs When…**	**Advantages**	**Limitations**
Complete-case analysis	Covariates Treatment Outcome	Missing data are MCAR.	MCAR	Straightforward	May be inefficient because of the loss in sample size
Last observation carried forward	Covariates Treatment Outcome (except at baseline)	The true, but missing, value is the same as the last available measurement *or* the treatment decision depends on the previous available measurement rather than the true (unobserved) one.	Constant	Straightforward Discards fewer patients than complete-case analysis	Can lead to confidence intervals that are too narrow Patients are discarded if baseline measurements are missing.
Multiple imputation	Covariates Treatment Outcome	Missing data are MAR.^a^ The imputation model is correctly specified.	MCAR MAR|*A*, *L* MAR|*A*, *L*, *Y* MAR|*A*, *L*, *V*	Maintains the original sample size	May be computationally intensive Challenging for a large number of time points
Inverse-probability-of-missingness weighting	Covariates Treatment Outcome	Missing data are MAR given the treatment and the covariates, but not the outcome. The weight model is correctly specified.	MCAR MAR|*A*, *L* MAR|*A*, *L*, *V* Constant	Faster than multiple imputation for large data setsWeights simultaneously address confounding and missing data.	May be inefficient for small and moderate sample sizes
Missingness pattern approach	Covariates	The partially observed covariate is no longer a confounder once missing (e.g., the treatment decision depends on the confounder value only when a measurement is available).	Differential	Relatively simple to implement Assumptions do not relate to Rubin’s taxonomy, so this method may work when standard methods do not.	Does not handle missing data on the exposure or outcome Challenging when the number of missingness patterns is large

Abbreviations: MAR, missing at random; MCAR, missing completely at random; MSM, marginal structural model.

^a^ Extensions with which to accommodate data that are missing not at random exist but are challenging to apply in practice.

## METHODS

**Figure 1:**
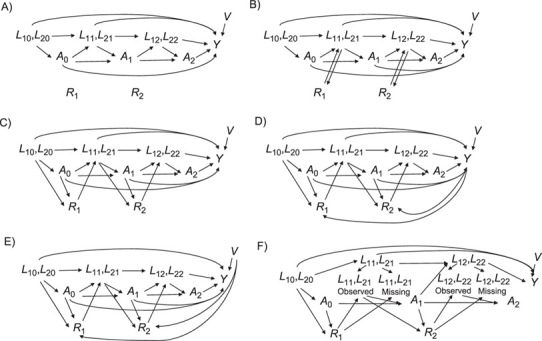
Causal graphs representing several possible scenarios for missing values. At each time point, *L*_1_ and *L*_2_ are the 2 time-varying confounders, *A* is the time-varying treatment, *Y* is the outcome, *V* is an independent risk factor, and *R* is the missingness indicator. The diagrams represent scenarios in which missingness occurs completely at random (MCAR) (A); occurs when there has been no change since the previous measurement (constant) (B); occurs at random given the confounders and treatment (MAR|*A*, *L*) (C); occurs at random given the confounders, treatment, and outcome (MAR|*A*, *L*, *Y*) (D); occurs at random given the confounders, treatment, and an independent risk factor (E); and depends on the treatment and confounders but the association between the missing value and the subsequent treatment allocation no longer exists (differential) (F). Under the panel F scenario, the confounder only contributes to the treatment allocation decision when it is observed. MAR, missing at random; MCAR, missing completely at random.

We performed a simulation study to 1) illustrate the impact on bias of violations of the assumptions required for each method to be valid, and the relative precision of these methods when assumptions hold, and 2) highlight existing challenges in their implementation in practice. Data were simulated to mimic an observational study evaluating the effect of a time-varying binary treatment on a continuous outcome in the presence of time-varying confounding. We focused on 4 plausible types of missingness mechanisms (Figure 1).

- MCAR mechanism: Missingness is not dependent on either observed variables or unobserved variables.
- MAR mechanism: We consider 3 situations—1) missing data depend on observed past treatment and confounder values (MAR|*A*, *L*); 2) missing data depend on past treatment and confounder values and outcome (MAR|*A*, *L*, *Y*); and 3) missing data are associated with the outcome through the independent risk factor (MAR|*A*, *L*, *V*).
- “Constant” mechanism: Confounder values are missing if they have remained constant since the last visit (a mechanism under which LOCF is expected to perform well).
- “Differential” mechanism: The missingness mechanism itself is MAR, but missingness affects the subsequent association between the true value of the confounder and the treatment (the mechanism implicitly assumed by the MPA). In other words, the past *observed* values of the confounders and treatment predict missingness, but among persons with a *missing* covariate value at a given time point, there is no association between the true (but unmeasured) value and the subsequent treatment received.

We compared the performance of CC analysis, LOCF, MPA, MI, and IPMW to estimate the causal effect of the intervention at each time point. The analysis model was the additive model proposed by Daniel et al. (3) and Robins et al. (7):

$$ Y={\beta}_{\mathrm{int}}+{\beta}_0{a}_0+{\beta}_1{a}_1+{\beta}_2{a}_2, $$

where *Y* is a continuous outcome, *a_k_* are the binary treatment indicators at time *k* (*k* = 0, 1, 2), and the β coefficients are the parameters of the MSM. This model allows estimation of the contrast between any treatment strategies of interest. The data-generating mechanisms, methods, estimands, and performance measures for our simulations are presented in Web Appendix 4, and the R code with which to generate the data is available in Web Appendix 5. In the main scenario, the proportion of missing data was around 40%, and the sample size was 10,000. We also investigated the impact of a smaller proportion of missing data (5%) and a smaller sample size (*n* = 500).

## RESULTS

**Figure 2:**
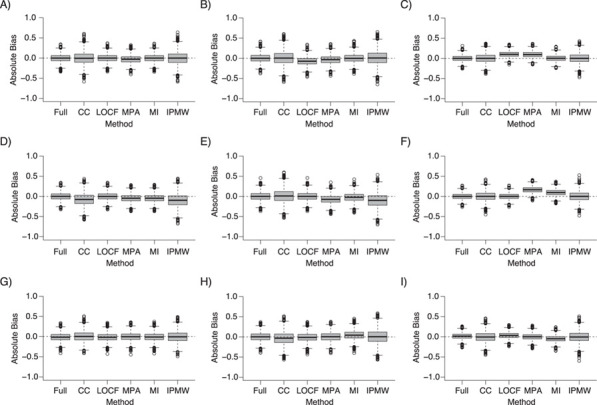
Absolute bias of the treatment effect estimate at *k* = 0 (left column (panels A, D, and G)), *k* = 1 (middle column (panels B, E, and H)), and *k* = 2 (right column (panels C, F, and I)) on full data and following the use of different missing-data approaches under missing completely at random (top row (panels A–C)), constant (middle row (panels D–F)), and differential (bottom row (panels G–I)) missingness mechanisms (*n* = 10,000; 40% of missing data). For multiple imputation, 10 imputed data sets were generated. CC, complete cases; IPMW, inverse-probability-of-missingness weighting; LOCF, last observation carried forward; MI, multiple imputation; MPA, missing pattern approach.

**Figure 3:**
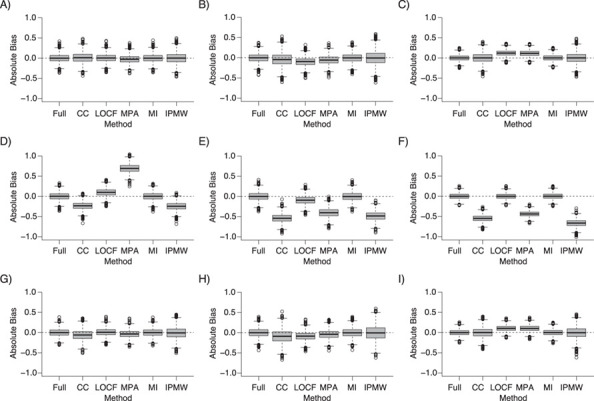
Absolute bias of the treatment effect estimate at *k* = 0 (left column (panels A, D, and G)), *k* = 1 (middle column (panels B, E, and H)), and *k* = 2 (right column (panels C, F, and I)) on full data and following the use of different missing-data approaches under 3 scenarios of data missing at random (MAR): 1) MAR given the covariates and the treatment (MAR|*A*, *L*) (top row (panels A–C)); 2) MAR given the covariates, the treatment, and the outcome (MAR|*A*, *L*, *Y*) (middle row (panels D–F)); and 3) MAR given the covariates, the treatment, and the independent risk factor (MAR|*A*, *L*, *V*) (bottom row (panels G–I)) (*n* = 10,000; 40% of missing data). For multiple imputation, 10 imputed data sets were generated. CC, complete cases; IPMW, inverse-probability-of-missingness weighting; LOCF, last observation carried forward; MI, multiple imputation; MPA, missing pattern approach.

The results of the main simulation study (*n* = 10,000 and 40% of missing data) are presented as box plots in Figures 2 and 3, showing the distribution of the absolute bias for each method, and are summarized in Table 1. Full results are presented in Web Tables 1–5 and Web Figures 1–4.

### Missingness completely at random

Whereas CC, MI, and IPMW lead to unbiased estimates at the 3 time points, the MPA estimates are biased at each time point and LOCF estimates are biased at times 1 and 2 (Figure 2). The bias for the MPA arises from the direct associations existing between the confounders and the treatment allocation at subsequent time points, even among participants with missing covariate values. For LOCF, the bias arises because the missing values were generally different from the observed previous value because confounder values were affected by prior treatment.

### Missingness at random

Except for MI, which led to unbiased estimates at each time point for the 3 MAR scenarios, the performance of the other analysis strategies relied on the variables that were predictive of missingness (Figure 3). When missingness depended on the values of past treatment assignment and confounders, IPMW estimates were unbiased at the 3 time points. A small bias was observed for CC analysis and larger biases were obtained when using LOCF and MPA, for similar reasons as in MCAR scenarios. When the outcome was directly related to missingness, the only unbiased approached was MI. However, when an indirect association between the outcome and missingness existed, the IPMW led to unbiased estimates, with a lower precision than MI.

### Missingness on constant values

Only LOCF was unbiased (Figure 3), and the bias was worse with MI and IPMW than with CC analysis. This is because they both use the existing relationships between the confounders, treatment, and outcome in the observed data, but in this scenario, these relationships do not reflect the associations existing between the true (missing) confounder values and the other variables.

### Missingness affecting the subsequent covariate-treatment associations

MPA was the only appropriate method for obtaining unbiased treatment effect estimates, although the bias of the other approaches was quite small. As in the previous scenario, the associations between confounders and treatment among the complete cases cannot be used to make inferences about the relationship among participants with missing confounder values. Therefore, CC analysis, MI, and IPMW are biased. LOCF estimates are unbiased when missingness in a variable depends only on the previous measurement for that variable. However, in the current scenario, missingness depends on past values of the treatment and confounders.

When the sample size was small (*n* = 500), the magnitude of bias was similar to that observed for the sample size of 10,000, but the standard errors of the treatment effect estimates were very large, illustrating the lack of efficiency of MSMs in small samples (Web Figures 3 and 4).

When only 5% of the data were missing, biases were smaller in magnitude and were, in our setting, negligible for non-MAR situations. However, we would not recommend the implementation of a method known to be biased when unbiased alternatives exist.

## ILLUSTRATIVE EXAMPLE

To illustrate the different methods, we analyzed data from a French prospective national cohort study that uses the research database of the Observatoire Sommeil de la Fédération de Pneumologie (OSFP). The OSFP registry is a standardized Web-based report containing anonymized longitudinal data on patients with sleep disorders (21). We aimed to estimate the causal effect of compliance with the use of a continuous positive airway pressure (CPAP) device on sleepiness symptoms among patients diagnosed with obstructive sleep apnea. In the OSFP registry, the number of recorded visits per patient varies, so for simplicity, we focused on patients who had follow-up visits within 3 months, 6 months, and 1 year after initiation of CPAP treatment in order to focus on the problem of missing records rather than sparse follow-up. Compliance was determined as use of a CPAP device for an average of 4 or more hours per night within each time interval. The outcome was a continuous sleepiness score measured during the last visit using the Epworth sleepiness scale (22). Age, sex, body mass index (weight (kg)/height (m)^2^), nocturia, and the presence of depression were considered as potential confounders in this study. The investigators intended to record updated values of body mass index, nocturia, and depression at each visit; however, measurement was not always undertaken as planned. Patients could have 1 (or more) missing measurement(s) on at least 1 of these variables. Patients’ characteristics are described in Web Table 6.

Out of 1,169 patients included, only 263 (22.8%) had complete records with no missing values (i.e., were complete cases) (Web Table 7). Data were not MCAR, since associations were observed between all potential confounders and the probability of having a complete record. However, an MAR mechanism was plausible in this setting. The MPA was not a suitable method for this analysis because of missing data on the CPAP exposure.

**Figure 4:**
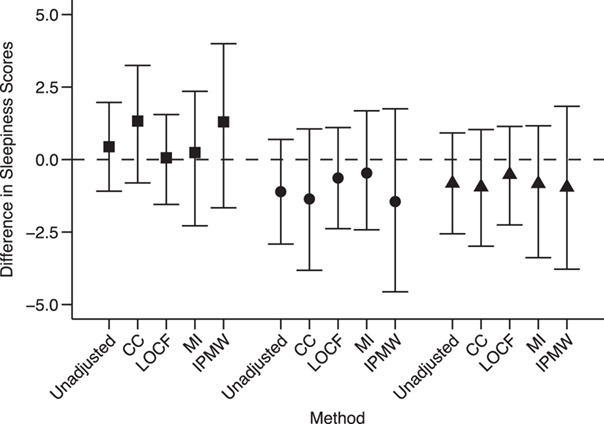
Results of applying unadjusted analysis, complete-case (CC) analysis, the last observation carried forward (LOCF) approach, multiple imputation (MI), and inverse-probability-of-missingness weighting (IPMW) to an illustrative example on sleep apnea using data from the Observatoire Sommeil de la Fédération de Pneumologie registry. Each point represents the difference in sleepiness scores at the end of the study (12 months) between compliers (use of a continuous positive airway pressure (CPAP) device for ≥4 hours/night) and noncompliers (CPAP use for <4 hours/night) at each of 3 time points (3 months (squares), 6 months (circles), and 12 months (triangles)). Bars, 95% confidence intervals.

Results are presented in Figure 4 and Web Table 8. Overall, we found no causal effect of CPAP compliance on sleepiness. Due to the relatively small sample size, IPMW led to very wide 95% confidence intervals. The 95% confidence interval for LOCF was narrower but did not account for the uncertainty around the imputed values. Furthermore, the assumption underlying the validity of LOCF was unlikely to hold here. As expected, all approaches produced similar results because confounding was not very strong (Web Table 7). However, this application illustrates the inefficiency of IPMW and the limitations of the MPA approach when exposure data are missing.

## DISCUSSION

In this paper, we have presented 5 methods for handling missing values in partially observed time-varying covariates in MSMs, identified situations in which they are appropriate for estimating unbiased causal effects, and illustrated how to implement these approaches in practice. We have shown that, for the estimation of causal effects, CC analysis is often biased, unless data are MCAR. The validity of this assumption cannot be tested from the data (23), but violations of these assumptions can be detected by looking at associations between the probability of being a complete case and the variables available in the data set. While the MCAR assumption is rarely plausible, we also showed that when missing values are MAR given treatment history and confounders, the bias of the CC estimates is usually small.

LOCF leads to biased estimations of the treatment effects, unless missing values are in truth missing because they remained constant over time or when the previous measurement is used to adapt treatment (rather than the true—but missing—measurement). This assumption may hold in routinely collected data. For instance, a general practitioner might not record a patient’s weight during a visit if it has not changed since the previous consultation. This assumption cannot be tested from the data, but the plausibility of the assumption can be assessed using expert opinion, building on what has been proposed in randomized trials (24). Moreover, when using LOCF, the uncertainty around the single imputation of missing values is not accounted for (25). Although this is not an issue for categorical variables, it is problematic for continuous confounders, where imputing exactly the previous measurement may lead to inappropriate certainty.

As expected, MI led to unbiased estimates of the treatment effect when data were MCAR or MAR. When implementing MI, the outcome must be included in the imputation model and the treatment effect estimated in each imputed data set and combined using Rubin’s rules, as recommended in settings with a single time point (26). In our simulations and example, treatment and covariate values at all time points were included in the imputation model. With an increasing number of time points, issues of overfitting may arise. Two-fold multiple imputation has been proposed for circumventing this problem (27). Instead of using all the time blocks in the imputation model, only the current and adjacent times are used, and therefore there are fewer parameters to be estimated in the imputation model.

MPA and IPMW have never been investigated in the context of MSMs. The MPA is unbiased when either the association between the partially observed confounder and the outcome or the association between the partially observed confounder and the subsequent treatment disappears among patients with a missing value for that confounder. Hence, the validity of this approach depends not on the missingness mechanism but instead on the relationship between covariates, treatment, and outcome among patients with missing data. In routinely collected primary-care data, this assumption is plausible when, for instance, the results of a blood test are used by the general practitioner to adapt the treatment prescription. If these results are missing (i.e., not available to the general practitioner), they will not be used in the treatment decision: Among patients with missing blood test results, the true (but unmeasured) value of the biological parameter is not directly associated with the treatment, and therefore the test result is no longer a confounder. Implementation of the MPA is straightforward, but issues may arise when there are many missingness patterns. The MPA’s inability to accommodate missing data on the treatment and the outcome might limit its applicability, unlike IPMW, which includes only the complete records in the analysis, regardless of which variables have missing values. IPMW generally leads to unbiased estimates when data are MCAR and MAR. The exception is the situation where missingness is directly affected by the outcome, because the outcome might be associated with treatment and confounder values at later time points, which are not accounted for in the missingness model used to compute the weights. IPMW is also unbiased in scenarios where the MPA is unbiased, because patients are censored at the first instance of missing data, and therefore no use is made of the information measured at later time points. However, IPMW is somewhat inefficient. This is explained by a loss in sample size and by the multiplication of 2 weights (the treatment weight and the missingness weight) that are both estimated with uncertainty, leading to highly variable treatment effect estimates. We recommend the use of IPMW in very large data sets—a situation in which MI would be highly computationally intensive. Furthermore, a limitation in the current implementation of IPMW is that missing data were considered monotonic; that is, patients were excluded from the analysis even when the outcome was available at the end of follow-up. Recent developments on inverse weighting have included an extension to nonmonotone missingness patterns (28), but it remains unclear how it could be transposed to MSMs.

It is clear that no single missing-data method can simultaneously handle different types of missingness mechanisms. However, in practice, missing values can occur in several variables according to different mechanisms. In such situations, it is crucial to understand the reasons for missingness to identify groups of variables with similar missingness mechanisms that could all be handled together. For instance, in routinely collected data, some variables might not have been updated because their values remained unchanged, and some variables might be missing at random. A pragmatic approach would be to first use the LOCF on the first group of variables and then multiply impute the variables from the second group. A more principled combination of methods has been proposed in simpler settings. Qu and Lipkovich (29) combined the MPA and MI for propensity score analysis with a single time point. Seaman et al. (30) proposed combining MI and IPMW, but further investigation is needed before these methods can be implemented in MSMs.

Although the role of our simulation was not to investigate the statistical properties of the 5 approaches in a broad range of settings but rather to empirically illustrate the theoretical findings, the design of the simulations had several limitations. First, we focused on a relatively simple setting with 3 time points and a few covariates. A plasmode simulation approach based on the sleep apnea study would have been more realistic but would not have allowed us to investigate the “constant” and “differential” mechanisms of missing data. Second, we generated data with a continuous outcome only. While this was chosen because bias is more easily observed with continuous outcomes, our conclusions will apply to binary and time-to-event outcomes; the validity of the methods relies on the missingness mechanism, which is independent of the nature of the outcome. Similar conclusions would also hold had the outcome been measured repeatedly. With a larger number of partially observed confounders, sparse data in some missingness patterns may preclude use of the MPA approach. Moreover, in the presence of numerous confounders with interactions and nonlinear effects, the functional form of the weight model might be harder to specify parametrically, and the obtained weights could be unstable. These problems may be alleviated by using more robust approaches (31) or statistical learning methods (32). Finally, the standard error of treatment effect estimates in our simulation study did not account for the uncertainty in the weight estimation, resulting in overly wide 95% confidence intervals. A nonparametric bootstrap was used in our illustrative example, but it was too computationally demanding for use in simulations.

In conclusion, the choice of the appropriate method(s) with which to handle partially observed confounders in MSMs must rely on careful consideration of the reasons for missingness and whether missingness modifies the existing relationships among observed data. Causal diagrams may help in understanding the structure of the data and the relationships between variables when data are missing and when data are observed. Although MI outperforms the other approaches when data are MAR, we presented 2 scenarios, encountered in routinely collected data, where MI leads to biased estimates of the treatment effect estimates but LOCF and the MPA might be suitable alternatives. Any analysis with missing data inevitably relies on assumptions about the missingness mechanisms or missingness patterns, which are often not made explicit. We therefore encourage researchers to clearly describe the assumptions under which their primary analysis is valid and to perform sensitivity analyses to assess the robustness of their results to departures from these postulated missingness mechanisms.

## Supplementary Material

Web_Material_kwaa225

## References

[1] Rubin DB. The design versus the analysis of observational studies for causal effects: parallels with the design of randomized trials. Stat Med. 2007;26(1):20–36. 17072897 10.1002/sim.2739

[2] Platt RW, Schisterman EF, Cole SR. Time-modified confounding. Am J Epidemiol. 2009;170(6):687–694. 19675141 10.1093/aje/kwp175 PMC2800260

[3] Daniel RM, Cousens SN, De Stavola BL, et al. Methods for dealing with time-dependent confounding. Stat Med. 2013;32(9):1584–1618. 23208861 10.1002/sim.5686

[4] Rosenbaum PR. The consequences of adjustment for a concomitant variable that has been affected by the treatment. J R Stat Soc Ser A. 1984;147(5):656–666.

[5] Robins JM. Marginal structural models. In: Proceedings of the American Statistical Association. (Section on Bayesian Statistical Science). Alexandria, VA: American Statistical Association; 1997:1–10.

[6] Cole SR, Hernán MA. Constructing inverse probability weights for marginal structural models. Am J Epidemiol. 2008;168(6):656–664. 18682488 10.1093/aje/kwn164 PMC2732954

[7] Robins JM, Hernán MA, Brumback B. Marginal structural models and causal inference in epidemiology. Epidemiology. 2000;11(5):550–560. 10955408 10.1097/00001648-200009000-00011

[8] Clare PJ, Dobbins TA, Mattick RP. Causal models adjusting for time-varying confounding—a systematic review of the literature. Int J Epidemiol. 2019;48(1):254–265. 10.1093/ije/dyy218 30358847

[9] Mojaverian N, Moodie EEM, Bliu A, et al. The impact of sparse follow-up on marginal structural models for time-to-event data. Am J Epidemiol. 2015;182(12):1047–1055. 26589708 10.1093/aje/kwv152 PMC4675663

[10] von Elm E, Altman DG, Egger M, et al. The Strengthening the Reporting of Observational Studies in Epidemiology (STROBE) statement: guidelines for reporting observational studies. J Clin Epidemiol. 2008;61(4):344–349. 18313558 10.1016/j.jclinepi.2007.11.008

[11] Karahalios A, Baglietto L, Carlin JB, et al. A review of the reporting and handling of missing data in cohort studies with repeated assessment of exposure measures. BMC Med Res Methodol. 2012;12:Article 96. 22784200 10.1186/1471-2288-12-96 PMC3464662

[12] Shortreed SM, Forbes AB. Missing data in the exposure of interest and marginal structural models: a simulation study based on the Framingham Heart Study. Stat Med. 2010;29(4):431–443. 20025082 10.1002/sim.3801

[13] Liu S-H, Chrysanthopoulou SA, Chang Q, et al. Missing data in marginal structural models: a plasmode simulation study comparing multiple imputation and inverse probability weighting. Med Care. 2019;57(3):237–243. 30664611 10.1097/MLR.0000000000001063 PMC6436551

[14] Moodie EEM, Delaney JAC, Lefebvre G, et al. Missing confounding data in marginal structural models: a comparison of inverse probability weighting and multiple imputation. Int J Biostat. 2008;4(1):Article 13. 22462119 10.2202/1557-4679.1106

[15] Vourli G, Touloumi G. Performance of the marginal structural models under various scenarios of incomplete marker’s values: a simulation study. Biom J. 2015;57(2):254–270. 25352223 10.1002/bimj.201300159

[16] Little R. Statistical Analysis With Missing Data. 2nd ed. Hoboken, NJ: John Wiley & Sons, Inc.; 2002.

[17] Blake HA, Leyrat C, Mansfield KE, et al. Propensity scores using missingness pattern information: a practical guide. Stat Med. 2020;39(11):1641–1657. 32103533 10.1002/sim.8503 PMC7612316

[18] Kreif N, Sofrygin O, Schmittdiel JA, et al. Evaluation of adaptive treatment strategies in an observational study where time-varying covariates are not monitored systematically [preprint]. ArXiv. 2018. (doi: 1806.11153) Accessed September 13, 2020.

[19] Hernán MA, Robins JM. Causal Inference: What If. Boca Raton, FL: Chapman & Hall/CRC Press; 2021.

[20] Sterne JAC, White IR, Carlin JB, et al. Multiple imputation for missing data in epidemiological and clinical research: potential and pitfalls. BMJ. 2009;338:b2393. 19564179 10.1136/bmj.b2393 PMC2714692

[21] Bailly S, Destors M, Grillet Y, et al. Obstructive sleep apnea: a cluster analysis at time of diagnosis. PLoS One. 2016;11(6):e0157318. 27314230 10.1371/journal.pone.0157318 PMC4912165

[22] Johns MW. A new method for measuring daytime sleepiness: the Epworth sleepiness scale. Sleep. 1991;14(6):540–545. 1798888 10.1093/sleep/14.6.540

[23] Potthoff RF, Tudor GE, Pieper KS, et al. Can one assess whether missing data are missing at random in medical studies? Stat Methods Med Res. 2006;15(3):213–234. 16768297 10.1191/0962280206sm448oa

[24] White IR, Carpenter J, Evans S, et al. Eliciting and using expert opinions about dropout bias in randomized controlled trials. Clin Trials. 2007;4(2):125–139. 17456512 10.1177/1740774507077849

[25] Molenberghs G, Thijs H, Jansen I, et al. Analyzing incomplete longitudinal clinical trial data. Biostatistics. 2004;5(3):445–464. 15208205 10.1093/biostatistics/5.3.445

[26] Leyrat C, Seaman SR, White IR, et al. Propensity score analysis with partially observed covariates: how should multiple imputation be used? Stat Methods Med Res. 2019;28(1):3–19. 28573919 10.1177/0962280217713032 PMC6313366

[27] Nevalainen J, Kenward MG, Virtanen SM. Missing values in longitudinal dietary data: a multiple imputation approach based on a fully conditional specification. Stat Med. 2009;28(29):3657–3669. 19757484 10.1002/sim.3731

[28] Sun B, Tchetgen Tchetgen EJ. On inverse probability weighting for nonmonotone missing at random data. J Am Stat Assoc. 2018;113(521):369–379. 30034062 10.1080/01621459.2016.1256814 PMC6051732

[29] Qu Y, Lipkovich I. Propensity score estimation with missing values using a multiple imputation missingness pattern (MIMP) approach. Stat Med. 2009;28(9):1402–1414. 19222021 10.1002/sim.3549

[30] Seaman SR, White IR, Copas AJ, et al. Combining multiple imputation and inverse-probability weighting. Biometrics. 2012;68(1):129–137. 22050039 10.1111/j.1541-0420.2011.01666.x PMC3412287

[31] Imai K, Ratkovic M. Robust estimation of inverse probability weights for marginal structural models. J Am Stat Assoc. 2015;110(511):1013–1023.

[32] Karim ME, Petkau J, Gustafson P, et al. On the application of statistical learning approaches to construct inverse probability weights in marginal structural Cox models: hedging against weight-model misspecification. Commun Stat Simul Comput. 2017;46(10):7668–7697.

