# Supplementary material for: Common Methods for Handling Missing Data in Marginal Structural Models: What Works and Why
Source: Am J Epidemiol. 2020 Oct 15;190(4):663–72. doi: 10.1093/aje/kwaa225 (PMC8631064; doi:10.1093/aje/kwaa225)
Supplement: Web_Material_kwaa225 [file Web_Material_kwaa225.zip › kwaa225 Leyrat Web Material Final.pdf]

Common Methods for Handling Missing Data in Marginal Structural Models:

What Works and Why

Clémence Leyrat[1*], James R. Carpenter[1,2], Sébastien Bailly[3], and Elizabeth J. Williamson[1,4]

Correspondence to Dr. Clémence Leyrat, Department in Medical Statistics, London School of Hygiene and Tropical Medicine, Keppel Street, WC1E 7HT London, United Kingdom.

Phone: +44 (0)207 927 2169; fax: +44 (0)20 7436 5389; e-mail: clemence.leyrat@lshtm.ac.uk.

Author affiliations:
Department of Medical Statistics, London School of Hygiene and Tropical Medicine, London, United Kingdom (Clémence Leyrat, James R. Carpenter, Elizabeth J. Williamson); London Hub for Trials Methodology Research, MRC Clinical Trials Unit, University College London, London, United Kingdom (James R. Carpenter); HP2, INSERM 1042, Université Grenoble Alpes, Grenoble, France (Sébastien Bailly); and Health Data Research, London, United Kingdom (Elizabeth J. Williamson).

Table of Contents
Web Appendix 1: The potential outcome framework and causal inference in single-time studies
Web Appendix 2: Estimating the weights of a marginal structural model
Web Appendix 3: Description of the missing data methods
Web Appendix 4: Design of the simulation study
Web Appendix 5: R code to generate the data
Web Tables 1–8
Web Figures 1–4

Web Appendix 1

The potential outcome framework and causal inference in single-time studies

Causal inference is often formalised using the potential outcomes framework (1), initially developed in the context of randomised experiments. In a simple setting in which one is interested in estimating the causal effect of a binary treatment A on an outcome Y measured at a single time point, the causal treatment effect is defined as the difference between the two potential outcomes $Y^{A=0}$ and $Y^{A=1}$. These are the outcomes that would have been observed had the population been treated versus not.

Three assumptions are usually made to consistently estimate the causal effect of a treatment: (i) no interference, (ii) consistency, and (iii) conditional exchangeability. Assumption (i) means that the potential outcome values for a given patient are not affected by the treatment values of other patients. Assumption (ii) implies that a patient has only one possible potential outcome value for each treatment under study and assumption (iii) ensures that there are no unmeasured confounders. If these assumptions hold, a multivariable regression model may allow the estimation of an unbiased conditional causal treatment effect, provided the model is correctly specified. More advanced statistical techniques, such as inverse-probability-of-treatment weighting (IPTW) may be used to estimate marginal causal effects. The validity of IPTW relies on the additional assumption of positivity (2), meaning that every patient has a non-null probability of receiving either treatment. IPTW aims to create a pseudo-population in which the treatment is independent of the confounders, analogous to the situation achieved by randomising the treatment in a trial, by weighting the patients by the inverse of their probability of receiving the treatment they actually received. This probability is derived from the propensity score (3) and can easily be estimated from the data using a logistic regression model. Both multivariable regression and IPTW are valid approaches to estimate causal effects (conditional and marginal, respectively) in a simple setting of a single time point. However, challenges arise when treatment and confounder values vary over time.

Web Appendix 2

Estimating the weights of a marginal structural model

Suppose there are K+1 measurement occasions, from k=0 (baseline) to the study end (k=K). Let A be a binary treatment, with A_k representing its level at time k (k=0,…,K) and $\bar{A}_k$ the treatment history until time *k*. L_k is a vector of confounders measured at time *k.* We assume that once a patient receives the treatment (A=1), they remain under treatment until the end of follow-up (k=K) where the outcome Y is measured. We are interested in the estimation of $E(Y^{\bar{a}})$, the value of the outcome that would have been observed had all subjects received treatment history $\bar{a}$. or contrasts of these quantities. For example, in our simulation study (Section 4 and 5), we estimate the effect of treatment initiation at each time point.

To estimate these quantities, the weights of the MSM are defined as the patient's probability of receiving their own treatment history given their health history. The stabilized weight for patient *i (i=1,…n)* is defined as:

$$sw_i = \frac{\prod_{k=0}^{K} P(A_k = a_{ki} | \bar{A}_{k-1} = \bar{a}_{(k-1)i})}{\prod_{k=0}^{K} P(A_k = a_{ki} | \bar{A}_{k-1} = \bar{a}_{(k-1)i}, \bar{L}_k = \bar{l}_{ki})}, \quad (1)$$

where *k=1,…,K* is the kth time point, A_k the treatment received at time k, $\bar{A}_k$ the treatment history until time *k*, L_k the covariates values at time *k* and $\bar{L}_k$ the covariates history until time *k*. Stabilized weights are preferred to standard weights (i.e. weights computed with 1 at the numerator), because they reduce the variability in treatment probabilities that may happen when some covariates are strong

predictors of treatment. In practice, both the numerator and denominator can be estimated using pooled logistic regression, not including and including confounder history, respectively. In pooled logistic regression, each person-time interval is considered as an observation. This pooled logistic regression model must include the confounders and their relevant interactions to ensure the distributions of confounders are balanced between treatment groups in the weighted pseudo-population at each time point.

Web Appendix 3

Description of the missing data methods

Complete case analysis. Complete case (CC) analysis is a straightforward and widely used approach to handle missing data in MSMs. In CC analysis, the parameters of the MSM are estimated from the sub-sample of patients with a complete record for all the variables and for all time points (4). The first limitation of this approach is a loss in sample size, which may affect the precision of the estimates. The second, – and more problematic – limitation, is the risk of invalid inference following its use. In standard regression modelling, CC analysis can allow the estimation of unbiased conditional estimates of the treatment effect provided that the probability of being a complete case is independent of the outcome (5). The validity of CC analysis to estimate marginal effects relies on the stronger assumption that missing data are MCAR (6). This assumption is rarely plausible, and its violations can usually be demonstrated using the observed data.

Multiple imputation. The general principle of MI is to generate multiple sets of plausible values for the missing observations by drawing from the posterior predictive distribution of these variables given the observed data. Multiple imputation of partially observed data sets leads to the generation of *M* complete data sets which are then analysed independently to produce *M* estimates $\hat{\theta}_k$, (*k = 1,…, M*) of θ, the vector of the parameters of interest (the regression coefficients for $A_0,\ldots,A_t$ in our setting) and their associated variance matrix $\boldsymbol{W_k}$. Then, $\hat{\theta}_k$and $\boldsymbol{W_k}$, are combined across the M imputed data sets following Rubin's rules in order to obtain an overall estimate and its variance, accounting for the noise introduced by the random elements of the imputation. These rules state that the overall estimate $\hat{\boldsymbol{\theta}}_{MI}$ and its estimated variance $\widehat{Var}(\hat{\theta}_{MI})$, are (7):

$$\hat{\boldsymbol{\theta}}_{MI} = \frac{1}{M}\sum_{k=1}^{M}\hat{\boldsymbol{\theta}}_k, \qquad \widehat{Var}(\hat{\boldsymbol{\theta}}_{MI}) = \boldsymbol{W} + \left(1 + \frac{1}{M}\right)\boldsymbol{B},$$

where $\boldsymbol{W}$ is the within-imputation covariance matrix, reflecting the variability in the estimates of the parameters of interest within each imputed data set and $\boldsymbol{B}$ is the between-imputation covariance matrix, reflecting how missing data impacts the variability in the estimates. These two variance components are estimated as:

$$\boldsymbol{W} = \frac{1}{M}\sum_{k=1}^{M}\boldsymbol{W_k}, \qquad \boldsymbol{B} = \frac{1}{M-1}\sum_{k=1}^{M}\left(\hat{\boldsymbol{\theta}}_k - \hat{\boldsymbol{\theta}}_{MI}\right)^2 \quad .$$

Because the confounders only enter the first stage of the MSM process, it may be tempting to combine the weights across imputed data sets, rather than combining the final treatment effect estimates. However, this approach is known to provide biased estimates (8).

MI can provide valid inference if the data are MAR, regardless of the role of variables predictive of missingness in the analysis model (i.e. treatment, outcome or confounder). Unfortunately, the

extensions of MI to accommodate data under MNAR mechanisms are limited (9). In addition, a correct specification of the imputation model is key to obtain valid inference after MI of the covariates. Critically, even when MI is used to handle missing values in confounder data, the outcome must be included in the imputation model in order to preserve the existing relationships between the variables and the outcome (10). Furthermore, in longitudinal studies, it has been recommended to include all the covariate measurements in the model as well as the full treatment history. In doing so, several measurements of the same variable are seen as independent variables, preserving the correlation between subsequent measurements. However, convergence problems may arise because of overfitting when the number of time points is large in comparison to the total sample size (11). Provided that factors associated with missingness are measured and the imputation model is correctly specified, MI may lead to unbiased estimates of the treatment effect (4) estimated from MSMs.

The missingness pattern approach. The missingness pattern approach (MPA) is a method proposed to handle missing data in the specific context of propensity-score analysis, including IPTW (12,13). This approach involves splitting the analysis sample into subsamples based on their missingness pattern. Then, the weight models are estimated separately within each pattern using only the variables fully observed in that pattern. When using IPTW with a single time point, the MPA can lead to the estimation of unbiased treatment effects under a set of assumptions which are not dependent on the missingness mechanism itself but rather on the existing relationships between the true (but unobserved) values of the confounders and the treatment and outcome (14,15). The first assumption is the absence of unmeasured confounding within each missingness pattern (which is the conditional exchangeability assumption introduced in Section 2.1 within each pattern). In addition, the MPA requires that the partially observed covariate is no longer a confounder once missing (for more details, see (14)). Because of the peculiarity of these assumptions, the MPA may be valid under some MNAR mechanisms but biased under some MCAR mechanisms. To our knowledge, this approach has not been extended yet to time-varying IPTW for MSMs, but might exhibit good performances provided the aforementioned assumptions hold at each time point.

The Last observation carried forward. Due to its simplicity, the last observation carried forward (LOCF) is another popular approach for handling missing data in studies with repeated measurements. LOCF is a form of single imputation, in which a missing covariate value is replaced by the most recent value recorded for that covariate for the same patient. The LOCF approach requires a complete measurement of the baseline data, because no prior information would be available to impute the first value. However, the main limitation of this approach is that its validity relies on the strong assumption that (i) either the true (but unobserved) distribution of the missing values at a given time point is exactly the same as the distribution of the observations used for the imputation (16) or (ii) when a value is missing, the treatment decision depends on the previous available measurement rather than the true (unobserved) one (17). In the first situation, LOCF is valid when the reason for missingness is the absence of change in values between two subsequent measurements. For instance, in routinely collected primary care data, a patient who is recorded as a non-smoker may not have smoking behaviour re-recorded unless they report a change (i.e. that they have started smoking). This is a very peculiar form of MNAR mechanisms where the associations between the missing confounder value and the previous observed value is deterministic. The second situation may also occur in routinely collected primary care data: general practitioners may base the treatment decision on the last available test results if the most recent results are not yet available. In the implementation of the LOCF approach, the uncertainty surrounding the imputation of missing values is not accounted for, leading to a potential underestimation of the variance of the treatment effect estimates, and therefore to an inflation of the type I error rate (18). MI can account for the uncertainty in the imputation but standard implementations of MI make the MAR assumption, so do not provide a direct alternative to LOCF when data follow this specific missingness mechanism.

Inverse-probability-of-missingness weighting. In MSMs, Robins and Hernán (19) proposed the use of censoring weights to account for patients lost to follow-up. Complete cases are re-weighted by the inverse of their probability of remaining in the study. Loss to follow-up can be viewed as a missing data problem, and therefore, these weights can be accommodated to account for missing data in an approach called inverse-probability-of-missingness weighting (IPMW). The main difference is that loss to follow-up will generate monotone missing data, that is, if data is missing at a given time point, it will be missing for all subsequent time points, whereas in MSMs, missing data are often sporadically missing. IPMW is very similar to IPTW introduced in section 2. Whereas IPTW aims to account for confounding by balancing the characteristics of treated and untreated patients, IPMW aims to additionally balance the characteristics of complete cases and incomplete cases (20). It thus involves the estimation of two sets of weights: treatment weights and missingness weights. The missingness weights are estimated using, for instance, equation (1) (Supplementary Material S2), but using R_k, the missingness indicator at time *k* (R_k=1 for complete cases and R_k=0 if at least one confounder has a missing value at time *k*). Under MCAR and MAR mechanisms, IPMW can allow the estimation of unbiased treatment effect estimates if the weight model is correctly specified (i.e. includes all the predictors of missingness in the correct functional form). The individual's missingness weights at time *t* are the probabilities of being a complete case up to that time.

The overall weight (i.e. the product of the weights of being treated and being a complete case) simultaneously deals with confounding and missing data. This approach was initially introduced in MSMs in the presence of loss to follow-up (19), meaning that, in our context of arbitrary missingness patterns, patients are censored once they have a missing confounder value, even when subsequent values are measured. Therefore, IPMW does not make use of all the information available, which can make it an inefficient technique for small to moderate sample sizes. However, IPMW might be preferred over MI when patients with missing data tend to have missing values on many, rather than just one or two, variables, as it is often the case in observational studies (20).

Web Appendix 4

Design of the simulation study

Aims

We performed a simulation study (i) to illustrate the impact on bias of violations of the assumptions required for each method to be valid, and the relative precision of these methods when the assumptions hold and (ii) to highlight existing challenges in their implementation in practice.

Data-generating mechanisms

Data were simulated to mimic an observational study, looking at the effect of a time-varying binary treatment on a continuous outcome, in the presence of time-varying confounding. Figure 1 presents a causal diagram illustrating the association between simulated variables. There are three measurement occasions, indexed by k=0,1,2. The continuous outcome was measured at the end of follow-up (*k*=2). Two time-varying confounders (one binary, L1, and one continuous, L2) were measured at baseline (*k*=0) and at two subsequent visits (*k*=1 and *k*=2) and one independent risk factor was measured at baseline only. The first treatment prescription was dependent on the baseline value of these 3 variables. The treatment prescription was then updated based on the current values of the two time-varying confounders and treatment history, creating a treatment-confounder feedback loop. For simplicity, we assumed that after treatment initiation, patients remained under treatment. The two time-varying confounders were fully observed at baseline, but were partially observed at the two subsequent visits. Patients could have either none, one or the two confounder values missing at visits 2 and 3. Missingness was arbitrary (i.e non-monotone); a patient could have a missing value at *k*=1

but a measured one at *k*=2. The data-generating mechanisms are described below and the R code to generate the data is provided as a supplementary file.

Under each scenario, around 40% of missing data were present at time 2 and 3, leading to a proportion of complete cases for all time points of about 40%.

Data generation procedure to obtain a complete data set
These steps allowed us to generate a complete data set before introducing missing data under a MCAR, MAR or "constant" mechanism. The data generation procedure was slightly different for the "differential" mechanism (see below): since missingness modifies the subsequent covariate-treatment relationships, missing values could not be introduced once the full data set was created.

Time *t*=0
Covariates:
L_1: binary time-varying covariate, $L_{10} \sim Bin(n, 0.5)$ at *t*=0
L_2: continuous time-varying covariate, $L_{20} \sim N(0,1)$ at *t*=0
V: independent time-fixed binary risk factor, $V \sim Bin(n, 0.3)$

Treatment allocation:
$A_0 \sim Bin(n, p_0)$ with $p_0 = expit(-1.5 + 0.6L_{10} + 0.3L_{20})$
Using this model, 24% of the patients received the treatment at the first time-point.

Time *t*=1
Potential covariate values at t=1 depending on the previous treatment:
L_{11_0}: value of L_1 if A_0=0 $L_{11\_0} \sim Bin(n, p_{L1})$ with $p_{L1} = expit(0.4L_{10})$
L_{11_1}: value of L_1 if A_0=1 $L_{11\_1} \sim Bin(n, p_{L2})$ with $p_{L2} = expit(0.4L_{10} + 0.4)$
L_{21_0}: value of L_2 if A_0=0 $L_{21\_0} = 0.5L_{20} + N(0, 0.1^2)$
L_{21_1}: value of L_2 if A_0=1 $L_{21\_1} = 0.2 + 0.5L_{20} + N(0, 0.1^2)$
The observed covariate value for each patient is the version of the covariate corresponding to the observed treatment.

Treatment allocation:
$A_1 = 1 \; if \; A_0 = 1$, otherwise:
$A_1 \sim Bin(n, p_1)$ with $p_1 = expit(-2.5 + 0.8L_{10} + 0.2L_{20})$
Using this model, 33% of the patients received the treatment at the second time-point. Note that once under treatment, patients cannot switch back to the control.

Time *t*=2
Potential covariate values at t=1 depending on the previous treatment:
L_{12_00}: value of L_1 if A_0=0 and A_1=0 $L_{12\_00} \sim Bin(n, p_{L120})$ with $p_{L120} = expit(0.4L_{11\_0})$
L_{12_01}: value of L_1 if A_0=0 and A_1=1 $L_{12\_01} \sim Bin(n, p_{L121})$ with $p_{L121} = expit(0.4L_{11\_0} + 0.4)$
L_{12_11}: value of L_1 if A_0=1 and A_1=1 $L_{12\_11} \sim Bin(n, p_{L122})$ with $p_{L122} = expit(0.4L_{11\_1} + 0.2)$
L_{22_00}: value of L_2 if A_0=0 and A_1=0 $L_{22\_00} = 0.5L_{20} + N(0, 0.1^2)$
L_{22_01}: value of L_2 if A_0=0 and A_1=1 $L_{22\_01} = 0.4 + 0.5L_{20} + N(0, 0.1^2)$
L_{22_11}: value of L_2 if A_0=1 and A_1=1 $L_{22\_11} = 0.2 + 0.5L_{20} + N(0, 0.1^2)$
The observed covariate values for each patient is the version of the covariate corresponding to the observed treatment history.

Treatment allocation:
$A_2 = 1\ if\ A_1 = 1$, otherwise:
$A_2 \sim Bin(n, p_2)$ with $p_2 = expit(-2 + 1L_{10} + 0.3L_{20})$
Using this model, 47% of the patients received the treatment at the third time-point.

Potential outcomes under the 4 possible treatment histories:
$Y_{000} \sim N(l_{000}, 2^2)$ with $l_{000} = 0.7L_{10} + 0.5L_{20} + L_{11\_0} + 0.7L_{21\_0} + 1.5L_{12\_00} + 1.5L_{22\_00} + V$
$Y_{001} \sim N(l_{001}, 2^2)$ with $l_{001} = 2 + 0.7L_{10} + 0.5L_{20} + L_{11\_0} + 0.7L_{21\_0} + 1.5L_{12\_00} + 1.5L_{22\_00} + V$
$Y_{011} \sim N(l_{011}, 2^2)$ with $l_{011} = 3 + 0.7L_{10} + 0.5L_{20} + L_{11\_1} + 0.7L_{21\_1} + 1.5L_{12\_01} + 1.5L_{22\_01} + V$
$Y_{111} \sim N(l_{111}, 2^2)$ with $l_{111} = 4 + 0.7L_{10} + 0.5L_{20} + L_{11\_1} + 0.7L_{21\_1} + 1.5L_{12\_11} + 1.5L_{22\_11} + V$

The observed outcome for a given patient is the one corresponding to the observed treatment history.
From these models, the true value of the regression parameters of this MSM (analysis model):

$$Y = \beta_{int} + \beta_0 a_0 + \beta_1 a_1 + \beta_2 a_2$$

are 1.1629, 1.67668 and 2

Introduction of missing data:
Let R_{1_1}, R_{2_1}, R_{1_2}, R_{2_2} the missingness indicators for variables L_1 and L_2 at time *t*=1 and 2, respectively. R=1 if the variable is observed, and R=0 if the variable is missing. The values of the parameters in the models below were chosen to ensure an overall proportion of missing data of around 40%.

MCAR mechanism
$R_{1\_1}, R_{2\_1}, R_{1\_2}, R_{2\_2}, \sim Bin(n, 0.2)$

MAR|A,L
At *t*=1
$R_{1\_1} \sim Bin(n, r_{1\_1})$ with $r_{1\_1} = expit(0.9 + 0.8L_{10} + 1.2A_0)$
$R_{2\_1} \sim Bin(n, r_{2\_1})$ with $r_{2\_1} = expit(1.4 + 0.3L_{10} + 0.7A_0)$

At *t*=2
For patients with a missing measurement at t=1, the probability of being missing at t=2 depends on the baseline measurement (to avoid generating MNAR data)

$R_{1\_2} \sim Bin(n, r_{1\_2})$ with
$r_{1\_2} = expit(0.85 + 0.6L_{11} + 1A_1)$ if L_{11} is observed
$r_{1\_2} = expit(0.85 + 0.6L_{10} + 1A_1)$ if L_{11} is missing

$R_{2\_2} \sim Bin(n, r_{2\_2})$ with
$r_{2\_2} = expit(1.2 + 0.3L_{21} + 1A_1)$ if L_{21} is observed
$r_{2\_2} = expit(1.2 + 0.3L_{20} + 1A_1)$ if L_{21} is missing

MAR|A,L,Y
At *t*=1
$R_{1\_1} \sim Bin(n, r_{1\_1})$ with $r_{1\_1} = expit(-0.2 + 0.8L_{10} + 1.2A_0 + 0.5Y)$
$R_{2\_1} \sim Bin(n, r_{2\_1})$ with $r_{2\_1} = expit(0.1 + 0.3L_{10} + 0.7A_0 + 0.5Y)$

At *t*=2

$R_{1\_2} \sim Bin(n, r_{1\_2})$ with
$r_{1\_2} = expit(0.6L_{11} + 1A_1 + 0.5Y)$ if L_{11} is observed
$r_{1\_2} = expit(0.6L_{10} + 1A_1 + 0.5Y)$ if L_{11} is missing

$R_{2\_2} \sim Bin(n, r_{2\_2})$ with
$r_{2\_2} = expit(0.2 + 0.3L_{21} + 1A_1 + 0.5Y)$ if L_{21} is observed
$r_{2\_2} = expit(0.2 + 0.3L_{20} + 1A_1 + 0.5Y)$ if L_{21} is missing

MAR|A,L,V
At *t*=1
$R_{1\_1} \sim Bin(n, r_{1\_1})$ with $r_{1\_1} = expit(0.6 + 0.8L_{10} + 1.2A_0 + 1.1V)$
$R_{2\_1} \sim Bin(n, r_{2\_1})$ with $r_{2\_1} = expit(0.85 + 0.3L_{10} + 0.7A_0 + 1.1V)$

At *t*=2
$R_{1\_2} \sim Bin(n, r_{1\_2})$ with
$r_{1\_2} = expit(0.75 + 0.6L_{11} + 1A_1 + 1.1V)$ if L_{11} is observed
$r_{1\_2} = expit(0.75 + 0.6L_{10} + 1A_1 + 1.1V)$ if L_{11} is missing

$R_{2\_2} \sim Bin(n, r_{2\_2})$ with
$r_{2\_2} = expit(0.9 + 0.3L_{21} + 1A_1 + 1.1V)$ if L_{21} is observed
$r_{2\_2} = expit(0.9 + 0.3L_{20} + 1A_1 + 1.1V)$ if L_{21} is missing

Constant mechanism
$R_{1\_1} \sim Bin(m, 0.5)$ where m is the number of patients for whom L_{10}=L_{11}
$R_{1\_2} \sim Bin(s, 0.5)$ where s is the number of patients for whom L_{11}=L_{12}
$R_{2\_1} = 1$ if abs(L_{21}-L_{20}<0.07)
$R_{2\_2} = 1$ if abs(L_{21}-L_{20}<0.05)

Differential mechanism
Missing data were introduced using the models described for the MAR mechanisms. However, instead of introducing missing data after the generation of a full data set, we introduced missing data at time t=1 before drawing the treatment allocation at time t=1 or the covariates at t=2. Similarly, missing data at t=2 where introduced before drawing the treatment allocation at time t=2.
Among patients with covariates observed at t=1, the model for treatment allocation is the same as described earlier. Among patients with missing covariates values, the true value of the covariate is not used in the model for treatment allocation. Therefore, the covariates are confounders when observed but not when they are missing.

Methods

Treatment effects estimates were obtained via MSM with IPTW as described in Supplementary material S2. At time point *k* (*k*=0,1,2), inverse-probability-of-treatment weights were estimated using a logistic regression model with the treatment received at time t as the outcome and the two confounders L_1 and L_2 at that time point as predictors. Only participants untreated at the previous time point contributed to the estimation of the weights since participants remain under treatment once they have initiated it. Consequently, individual weights stayed constant after initiation. The final weights used for the analysis of the primary outcome were the product of the weights up to the time

of initiation or up to time 2, whichever occurred first. The outcome model was a weighted linear regression model including binary treatment indicators at the three time points.

We compared the following methods to handle missing data:

- CC analysis: the parameters of the MSM are estimated using complete cases only, that is patients with a measurement available for each of the two time-varying confounders at each of the three time points.
- LOCF: when a patient has a missing confounder value at time *k,* it is imputed by the most recent value observed for that confounder. These singly-imputed values are then treated as if they were the observed values; thus, the treatment effect estimates are obtained using a MSM from this single imputed data set.
- MI: missing confounders values are imputed 10 times using multiple imputation by chained equations. The imputation model includes the treatment indicator and confounder values at the three time points, as well as the independent risk factor and the outcome. The MSM is fitted in each imputed data set and the 10 treatment effect estimates are pooled using Rubin's rules.
- MPA: at times *k*=1 and *k*=1, missing data could occur on L_1 or L_2, leading to 4 missingness patterns at each of these time points. A different weight model is used within each pattern, including only the variables fully observed in that pattern. Therefore, weights are estimated for every patient based on available information, and the analysis model is fitted on the entire original sample.
- IPMW: the weights for being a complete case are estimated at each time point. At baseline (k=0), there is no missing data, so the weight is 1 for everyone. Then, for *k*=1 and *k=2*, the weights are estimated as follows: first, a logistic regression model is fitted separately for each time point. The outcome is the binary missingness indicator at that time point, and the predictors are the independent risk factor, and the treatment and confounders history. The weights at the 3 time points are then multiplied together to obtain an overall weight for being a complete case, itself multiplied by the overall treatment weight (estimated as in the CC analysis). In the second stage (the analysis model to obtain the MSM parameters), complete cases were re-weighted using the pooled weights (instead of the treatment weights).

Estimands

The three estimands of interest were the causal mean differences in the continuous outcome between treated and untreated participants at time *k*: $\hat{\theta}_0$ is the direct effect of the treatment A_0, unmediated by A_1 and A_2. Similarly, $\hat{\theta}_1$ is the direct effect of A_1, unmediated by A_2 and $\hat{\theta}_2$ is the direct effect of A_2.

Sample size and number of simulations

The sample size was 10,000 in each generated data set for the main scenario, but we also considered n=500 as a sensitivity analysis. In the main scenario, 40% of missing data were introduced but we also considered a scenario with only 5% of missing data5000 replications were used.

Performance measures

The performance of the five methods was assessed using the following measure:

- Bias of the treatment effect estimate at each time point *k (k=0,1,2)*: $B_k = E(\hat{\theta}_k) - \theta_k$ estimated as: $\frac{1}{5000}\sum_1^{5000} \hat{\theta}_{ki} - \theta_k$, where θ_k is the true value of the treatment effect at time *k* and $\hat{\theta}_{ki}$ the estimate of the treatment effect in the *i^th^* simulated data set (i=1,…,5000).

- The empirical standard error of the treatment effect estimate at time *k*: $SE_k = \sqrt{Var(\hat{\theta})}$, estimated from the data as: $\sqrt{\frac{1}{4999}\sum_{i=1}^{5000}\left(\hat{\theta}_{ki} - \hat{\theta}_k\right)^2}$, where $\hat{\theta}_k$ is the average treatment effect at time *k* across the 5000 simulated data sets.
- The coverage rate, defined as the proportion of 95% confidence intervals containing the true value of the treatment effect.

The Monte-Carlo standard errors for these measures were also computed, as suggested by Morris *et al.* (21). All simulations were performed using R 3.5.1. The *mice* package (22) was used for multiple imputation using chained equations and the package *Survey* was used to conduct the weighted linear regression analysis (23).

Web Tables 1–8

Web Table 1. Absolute bias and coverage rate (%) before the generation of missing values, for unadjusted, covariate adjusted and MSM analyses

Method	A0		A1		A2	
	Bias	*Coverage*	*Bias*	*Coverage*	*Bias*	*Coverage*
Unadjusted	0.331	9.6	-0.187	61.6	0.462	0.0
Multivariable adjustment	-0.229	29.1	-0.592	0.0	0.003	94.8
MSM	0.000	97.2	0.001	97.4	0.000	97.9

n=10000. The unadjusted analysis does not account for covariates other than treatment history. With multivariable adjustment, confounders at each time point are included in the outcome model. MSM: marginal structural model. 5000 simulations were performed. The maximum Monte-Carlo standard error was 0.002 for the bias and 0.005 for the coverage rate.

Web Table 2. Absolute bias and coverage rate (%) for the 5 methods to handle missing data in each scenario considered at time 0

Scenario	CC		LOCF		MPA		MI		IPMW	
	Bias	*Coverage*	*Bias*	*Coverage*	*Bias*	*Coverage*	*Bias*	*Coverage*	*Bias*	*Coverage*
MCAR	-0.003	97.6	0.000	97.5	-0.030	96.3	-0.001	97.3	-0.004	96.4
MAR|AL	0.010	97.2	0.004	97.2	-0.029	96.3	0.000	97.0	0.000	96.6
MAR|ALY	-0.234	36.3	0.099	88.8	0.694	0.0	0.002	97.1	-0.245	36.3
MAR|ALV	-0.060	95.5	0.009	97.7	-0.034	96.5	0.000	97.7	-0.008	96.4
Constant	-0.074	95.7	0.000	97.1	-0.046	95.5	-0.045	95.8	-0.098	93.2
Differential	0.006	97.3	-0.013	97.3	-0.002	97.0	-0.007	97.3	-0.005	96.9

CC: complete cases; LOCF: last observation carried forward; MPA: missing pattern approach; MI: multiple imputation; IPMW: inverse probability of missingness weighting. The initial sample size was n=10000. After introduction of missing data, the average number of complete cases varied between n=4096 and n=5698, depending on the scenario. For multiple imputation, 10 imputed data sets were generated. 5000 simulations were performed. The maximum Monte-Carlo standard error was 0.002 for the bias and 0.005 for the coverage rate.

Web Table 3. Absolute bias and coverage rate (%) for the 5 methods to handle missing data in each scenario considered at time 1

Scenario	CC		LOCF		MPA		MI		IPMW	
	Bias	*Coverage*	*Bias*	*Coverage*	*Bias*	*Coverage*	*Bias*	*Coverage*	*Bias*	*Coverage*
MCAR	0.005	96.9	-0.074	93.7	-0.036	96.1	-0.001	97.4	0.006	96.3
MAR|AL	-0.043	97.0	-0.096	89.8	-0.055	95.2	-0.003	97.1	-0.005	97.0
MAR|ALY	-0.540	0.1	-0.093	92.6	-0.404	25.4	-0.002	98.1	-0.485	1.9
MAR|ALV	-0.089	94.7	-0.085	91.8	-0.039	96.0	-0.001	97.0	-0.004	96.8
Constant	0.012	97.9	0.000	97.4	-0.072	93.5	-0.021	97.1	-0.100	94.4
Differential	-0.032	97.5	-0.009	97.3	0.003	96.9	0.045	96.0	0.006	97.1

CC: complete cases; LOCF: last observation carried forward; MPA: missing pattern approach; MI: multiple imputation; IPMW: inverse probability of missingness weighting. The initial sample size was n=10000. After introduction of missing data, the average number of complete cases varied between n=4096 and n=5698, depending on the scenario. For multiple imputation, 10 imputed data sets were generated. 5000 simulations were performed. The maximum Monte-Carlo standard error was 0.002 for the bias and 0.005 for the coverage rate.

Web Table 4. Absolute bias and coverage rate (%) for the 5 methods to handle missing data in each scenario considered at time 2

Scenario	CC		LOCF		MPA		MI		IPMW	
	Bias	*Coverage*	*Bias*	*Coverage*	*Bias*	*Coverage*	*Bias*	*Coverage*	*Bias*	*Coverage*
MCAR	0.000	98.1	0.100	80.2	0.094	82.8	0.002	97.9	0.000	97.0
MAR|AL	0.002	98.1	0.122	71.3	0.115	75.6	0.004	97.7	0.002	97.2
MAR|ALY	-0.547	0.0	0.000	98.7	-0.437	0.0	0.002	98.3	-0.663	0.0
MAR|ALV	-0.002	97.9	0.104	79.3	0.103	79.7	0.001	97.8	-0.003	96.8
Constant	0.001	98.1	0.001	97.8	0.165	49.9	0.095	83.8	0.001	97.6
Differential	-0.004	97.9	0.034	96.2	0.001	96.8	-0.048	94.9	-0.003	96.9

CC: complete cases; LOCF: last observation carried forward; MPA: missing pattern approach; MI: multiple imputation; IPMW: inverse probability of missingness weighting. The initial sample size was n=10000. After introduction of missing data, the average number of complete cases varied between n=4096 and n=5698, depending on the scenario. For multiple imputation, 10 imputed data sets were generated. 5000 simulations were performed. The maximum Monte-Carlo standard error was 0.002 for the bias and 0.005 for the coverage rate.

Web Table 5. Mean squared error of the mean differences for the different methods

	A0						A1						A2					
	**Full**	**CC**	**LOCF**	**MPA**	**MI**	**IPMW**	**Full**	**CC**	**LOCF**	**MPA**	**MI**	**IPMW**	**Full**	**CC**	**LOCF**	**MPA**	**MI**	**IPMW**
MCAR	0.009	0.023	0.009	0.010	0.009	0.025	0.011	0.028	0.016	0.013	0.012	0.030	0.005	0.013	0.015	0.014	0.006	0.014
MARAL	0.009	0.016	0.009	0.010	0.010	0.019	0.011	0.027	0.020	0.015	0.012	0.028	0.005	0.014	0.020	0.019	0.006	0.016
MARALY	0.009	0.063	0.019	0.492	0.009	0.070	0.010	0.303	0.019	0.175	0.011	0.248	0.005	0.306	0.005	0.196	0.005	0.447
MARALV	0.009	0.020	0.009	0.010	0.009	0.020	0.011	0.034	0.018	0.013	0.012	0.033	0.005	0.014	0.016	0.016	0.006	0.020
Constant	0.009	0.028	0.009	0.011	0.011	0.035	0.011	0.026	0.011	0.016	0.012	0.040	0.005	0.012	0.005	0.033	0.015	0.014
Differential	0.009	0.017	0.010	0.010	0.010	0.019	0.011	0.026	0.012	0.013	0.014	0.029	0.006	0.015	0.007	0.006	0.008	0.018

Web Table 6. Description of the exposure groups at baseline (illustrative example)

	**Non compliant**	**Compliant**	**Standardised mean difference (%)**
	n*=162	n*=882	
Female	64 (39.5)	286 (32.4)	14.8
Age (mean (SD))	55.4 (13.5)	57.7 (12.9)	17.2
Sedentary	20 (12.4)	160 (18.2)	16.0
BMI (mean (SD))	32.1 (6.9)	32.0 (6.2)	1.1
Depression score** (mean (SD))	4.5 (3.9)	4.3 (3.9)	5.6
Frequency of urination>1	71 (72.4)	347 (77.5)	11.6

* Depression score was calculated using the Pichot depression scale

Web Table 7. Description of the sample (illustrative example) and proportions of missing data (*n* = 1,169)

**Variable**	**T0**		**T1**		**T3**		**Over the full period**
	**n (%)**	**Missing n(%)**	**n (%)**	**Missing n(%)**	**n (%)**	**Missing n(%)**	**Missing at least one measurement n(%)**
**Compliance**	882 (84.5)	125 (10.7)	931 (87.3)	102 (8.7)	938 (86.9)	90 (7.7)	272 (23.3)
**Sex**	382 (32.7)	0 (0.0)	-	-	-	-	0 (0.0)
**Age (mean (SD))**	57.3 (12.9)	7 (0.6)	-	-	-	-	7 (0.6)
**Sedentarity**	205 (17.6)	4 (0.3)	-	-	-	-	4 (0.3)
**BMI (mean (SD))**	31.8 (6.2)	10 (0.9)	31.9 (6.2)	117 (10.0)	31.9 (6.0)	83 (7.1)	163 (13.9)
**Depression score* (mean (SD))**	4.4 (3.9)	85 (7.3)	2.8 (3.5)	164 (14.0)	2.5 (3.3)	153 (13.1)	257 (22.0)
**Nocturia**	465 (76.0)	557 (47.6)	302 (56.8)	637 (54.5)	265 (53.1)	670 (57.3)	739 (63.2)
**Overall**	-	648 (55.4)	-	761 (65.1)	-	765 (65.4)	903 (77.2)

* Depression score was calculated using the Pichot depression scale

Web Table 8. Estimated mean differences in sleepiness scores for the different analysis methods

**Analysis method**	**n**	**Mean difference (95% confidence interval)**		
		**t=1**	**t=2**	**t=3**
Unadjusted	897	-0.68 [-1.58;-0.21]	0.15 [-0.87;1.17]	-1.32[-2.28;-0.37]
IPTW				
Complete cases	266	-0.26 [-2.01;1.50]	0.27 [-2.20;2.74]	-0.07 [-2.41;2.26]
LOCF	521	-0.54 [-2.00;0.93]	-0.13 [-2.01;1.76]	-0.37 [-1.83;1.09]
Multiple imputation	1169	-0.80 [-1.77;0.16]	-0.13 [-1.29;1.04]	-1.19 [-2.20;-0.19]
IPMW	266	-0.38 [-2.24;1.47]	0.12 [-2.37; 2.60]	0.05 [-2.22;2.32]
MPA		NA	NA	NA

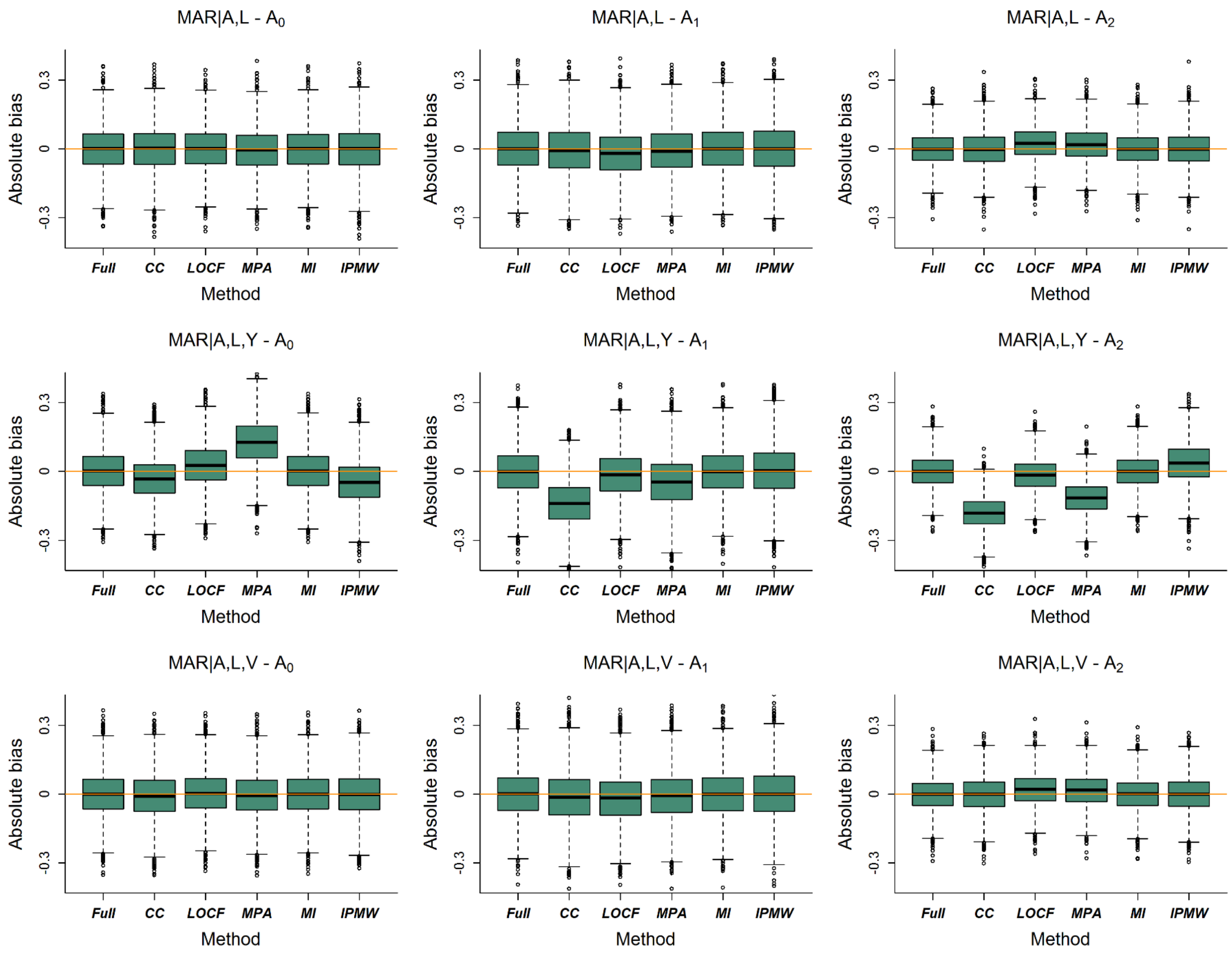

Web Figure 1. Absolute bias of the treatment effect estimate at k=0, k=1 and k=2 on full data and following the use of different missing data approach under the missing completely at random (MCAR), Constant and Differential missingness mechanisms. N=10000. 5% of missing data.

CC: complete cases; LOCF: last observation carried forward; MPA: missing pattern approach; MI: multiple imputation; IPMW: inverse probability of missingness weighting. For multiple imputation, 10 imputed data sets were generated.

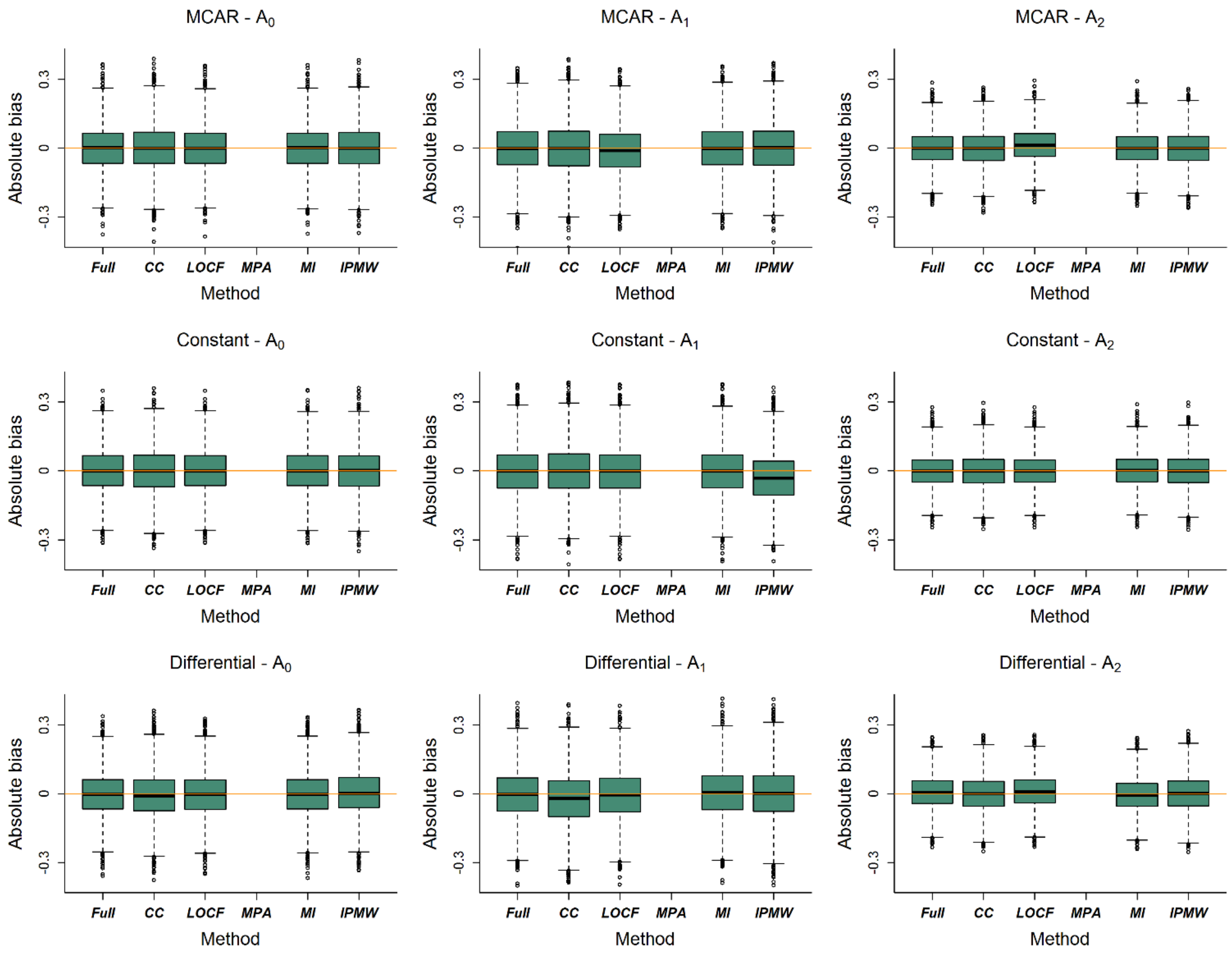

Web Figure 2. Absolute bias of the treatment effect estimate at k=0, k=1 and k=2 on full data and following the use of different missing data approach under the missing completely at random (MCAR), Constant and Differential missingness mechanisms. N=10000. 5% of missing data.

MPA estimates could not be obtained because the number of individuals with both L1 and L2 missing was too small for the model to converge.

CC: complete cases; LOCF: last observation carried forward; MPA: missing pattern approach; MI: multiple imputation; IPMW: inverse probability of missingness weighting. For multiple imputation, 10 imputed data sets were generated.

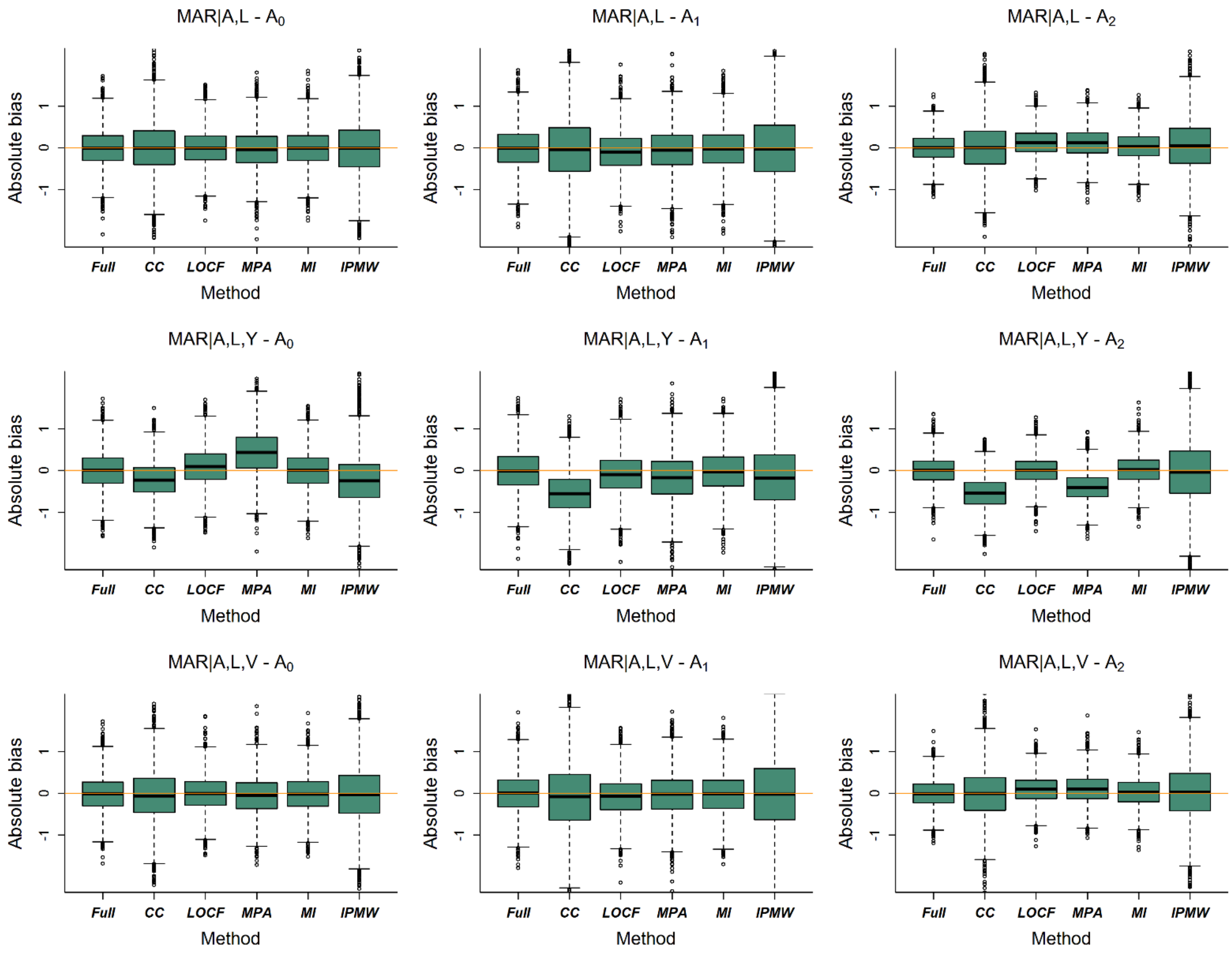

Web Figure 3. Absolute bias of the treatment effect estimate at k=0, k=1 and k=2 on full data and following the use of different missing data approach under the missing completely at random (MCAR), Constant and Differential missingness mechanisms. N=500. 40% of missing data.

CC: complete cases; LOCF: last observation carried forward; MPA: missing pattern approach; MI: multiple imputation; IPMW: inverse probability of missingness weighting. For multiple imputation, 10 imputed data sets were generated.

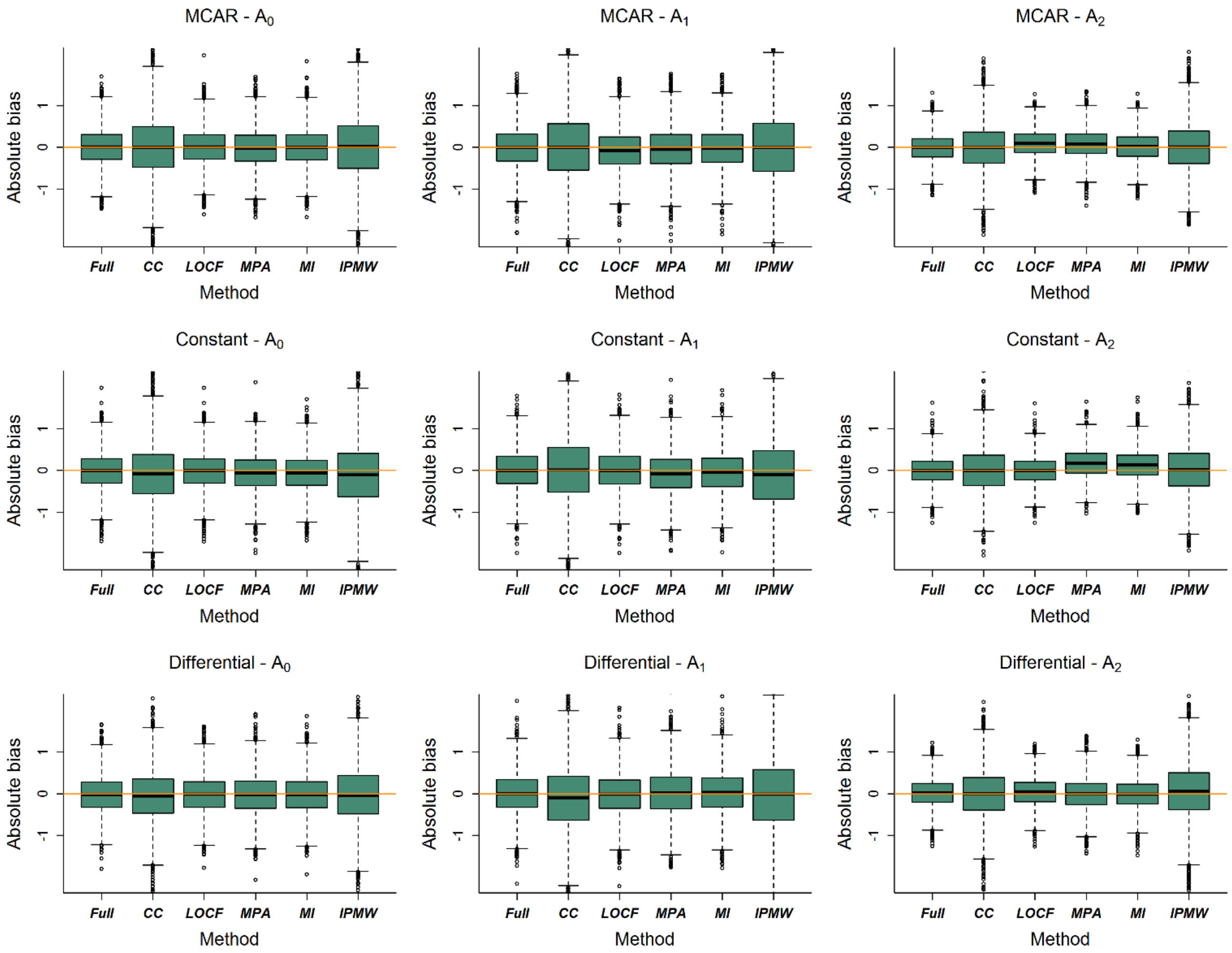

Web Figure 4. Absolute bias of the treatment effect estimate at k=0, k=1 and k=2 on full data and following the use of different missing data approach under the missing completely at random (MCAR), Constant and Differential missingness mechanisms. N=500. 40% of missing data.

CC: complete cases; LOCF: last observation carried forward; MPA: missing pattern approach; MI: multiple imputation; IPMW: inverse probability of missingness weighting. For multiple imputation, 10 imputed data sets were generated.

1. Rubin DB. Estimating causal effects of treatments in randomized and nonrandomized studies. J Educ Psychol. 1974;66(5):688–701.

2. Cole SR, Hernán MA. Constructing inverse probability weights for marginal structural models. Am J Epidemiol. 2008 Sep 15;168(6):656–64.

3. ROSENBAUM PR, RUBIN DB. The central role of the propensity score in observational studies for causal effects. Biometrika. 1983 Avril;70(1):41–55.

4. Moodie EEM, Delaney JAC, Lefebvre G, Platt RW. Missing Confounding Data in Marginal Structural Models: A Comparison of Inverse Probability Weighting and Multiple Imputation. Int J Biostat [Internet]. 2008 [cited 2017 May 22];4(1). Available from: https://www.degruyter.com/view/j/ijb.2008.4.1/ijb.2008.4.1.1106/ijb.2008.4.1.1106.xml

5. Bartlett JW, Harel O, Carpenter JR. Asymptotically Unbiased Estimation of Exposure Odds Ratios in Complete Records Logistic Regression. Am J Epidemiol. 2015 Oct 15;182(8):730–6.

6. Molenberghs G, Thijs H, Jansen I, Beunckens C, Kenward MG, Mallinckrodt C, et al. Analyzing incomplete longitudinal clinical trial data. Biostat Oxf Engl. 2004 Jul;5(3):445–64.

7. Carpenter J, Kenward M. Multiple Imputation and its Application. John Wiley & Sons; 2012. 299 p.

8. Leyrat C, Seaman SR, White IR, Douglas I, Smeeth L, Kim J, et al. Propensity score analysis with partially observed covariates: How should multiple imputation be used? Stat Methods Med Res. 2017 Jan 1;962280217713032.

9. Galimard J-E, Chevret S, Protopopescu C, Resche-Rigon M. A multiple imputation approach for MNAR mechanisms compatible with Heckman's model. Stat Med. 2016;35(17):2907–20.

10. Moons KGM, Donders RART, Stijnen T, Harrell FE. Using the outcome for imputation of missing predictor values was preferred. J Clin Epidemiol. 2006 Oct;59(10):1092–101.

11. Welch CA, Petersen I, Bartlett JW, White IR, Marston L, Morris RW, et al. Evaluation of two-fold fully conditional specification multiple imputation for longitudinal electronic health record data. Stat Med. 2014 Sep 20;33(21):3725–37.

12. Rosenbaum PR, Rubin DB. Reducing Bias in Observational Studies Using Subclassification on the Propensity Score. J Am Stat Assoc. 1984 Sep;79(387):516–24.

13. D'Agostino RB, Rubin DB. Estimating and Using Propensity Scores with Partially Missing Data. J Am Stat Assoc. 2000 Sep;95(451):749–59.

14. Blake HA, Leyrat C, Mansfield KE, Seaman S, Tomlinson LA, Carpenter J, et al. Propensity scores using missingness pattern information: a practical guide. ArXiv190103981 Stat [Internet]. 2019 Jan 13 [cited 2019 Feb 25]; Available from: http://arxiv.org/abs/1901.03981

15. Mattei A. Estimating and using propensity score in presence of missing background data: an application to assess the impact of childbearing on wellbeing. Stat Methods Appl. 2008 Jan 4;18(2):257–73.

16. Lachin JM. Fallacies of last observation carried forward analyses. Clin Trials Lond Engl. 2016 Apr;13(2):161–8.

17. Kreif N, Sofrygin O, Schmittdiel JA, Adams A, Grant R, Zhu Z, et al. Evaluation of adaptive treatment strategies in an observational study where time-varying covariates are not monitored systematically. arXiv:180611153. 2018;1806.11153.

18. Little RJA, Rubin DB. Single Imputation Methods. In: Statistical Analysis with Missing Data [Internet]. John Wiley & Sons, Ltd; 2014 [cited 2019 Mar 8]. p. 59–74. Available from: https://onlinelibrary.wiley.com/doi/abs/10.1002/9781119013563.ch4

19. Robins JM, Hernán MÁ, Brumback B. Marginal Structural Models and Causal Inference in Epidemiology. Epidemiology. 2000 Sep;11(5):550–60.

20. Seaman SR, White IR. Review of inverse probability weighting for dealing with missing data. Stat Methods Med Res. 2013 Jun 1;22(3):278–95.

21. Morris TP, White IR, Crowther MJ. Using simulation studies to evaluate statistical methods. Stat Med [Internet]. [cited 2019 Feb 22];0(0). Available from: https://onlinelibrary.wiley.com/doi/abs/10.1002/sim.8086

22. van Buuren S, Groothuis-Oudshoorn K. mice: Multivariate Imputation by Chained Equations in R. J Stat Softw. 2011;45(3):1–67.

23. Lumley T. Analysis of complex survey samples. J Stat Softw. 2004;9(1):1–19.
